# Evaluation of Organic Acids and Ultrasound as Pretreatment in Convective Drying Kinetics and Quality Parameters of Pumpkin

**DOI:** 10.3390/foods13162502

**Published:** 2024-08-09

**Authors:** José R. R. de O. Moura, Blenda R. S. de Morais, João H. F. da Silva, Amanda S. S. Alves, Shirley C. R. Brandão, Patricia M. Azoubel

**Affiliations:** 1 Departamento de Engenharia Química, Universidade Federal de Pernambuco, Av. Prof. Arthur de Sá, s/n, Cidade Universitária, Recife 50740-521, PE, Brazil; jose.roberto1996@yahoo.com.br (J.R.R.d.O.M.); blenda.morais@ufpe.br (B.R.S.d.M.); joao.hfsilva@ufpe.br (J.H.F.d.S.); 2 Departamento de Nutrição, Universidade Federal de Pernambuco, Av. Moraes Rego, s/n, Cidade Universitária, Recife 50670-901, PE, Brazil; amandassuellen@gmail.com; 3 Department of Food Science and Technology, College of Food, Agricultural, and Environmental Sciences, The Ohio State University, Columbus, OH 43210, USA; rupertbrandao.1@osu.edu

**Keywords:** pumpkin, organic acid, ultrasound, drying

## Abstract

There is a growing interest in the food industry in new drying technologies that reduce the time required for dehydration, combined with low energy consumption, low environmental impact, and maintenance of the overall quality of the product. This work investigated convective drying of pumpkin with and without ultrasound-organic (citric or acetic) acid pretreatment for different durations (10, 20, and 30 min). Drying was carried out at 60 °C, and the Wang and Singh model had the best fit for the experimental data. Samples pretreated for 30 min had the shortest drying times. Water diffusivities ranged from 6.68 × 10^−8^ m^2^/s to 7.31 × 10^−8^ m^2^/s, with the pretreated samples presenting the highest values. The dried pumpkin water activity values were below 0.60. Regarding color parameters, there was a slight increase in luminosity, a slight reduction in a*, and a significant increase in b*. Drying resulted in the loss of ascorbic acid and phenolic compounds, but the samples pretreated with citric acid showed better retention. There was also a reduction in the total carotenoid content, but samples pretreated with acetic acid for 10 and 20 min showed the best retention.

## 1. Introduction

The pumpkin is a popular name that is attributed to several species of vegetables in the Cucurbitaceae family, where the species *Cucurbita moschata*, *Cucurbita maxima*, and *Cucurbita pepo* stand out for their economic importance. It can grow in tropical and subtropical regions [1] and is rich in carotenoids, mineral salts, phenolic compounds, and vitamins, among others, which are essential for human nutrition. The vegetable has a very attractive color and is affordable, but it is a perishable food [2,3].

Drying is a unitary operation widely used in the preservation of food, and among different methods, convective drying is the one most used on plant materials. However, hot air can lead to negative aspects, as it can alter characteristics of the food, such as color, appearance, texture, and nutritional composition, among other parameters linked to the quality of the food, which, when dried, ends up displeasing the consumer [4]. Some of the consequences of drying are non-enzymatic browning (color and appearance) and degradation of nutritional components that are more sensitive to heat and thermodynamically unstable, such as carotenoids. Furthermore, convective drying leads to high energy consumption [5]. 

The use of pretreatments to reduce drying time and quality damage, such as ultrasound, has been studied. The application of ultrasound causes fluid cavitation, an energy phenomenon that disrupts the structure of food, creating new pores and channels within it. Through a variety of processes, including inertial flow, acoustic stirring, and microjets, this effect enhances mass and heat transmission [6,7]. 

Another pretreatment alternative for drying is the use of process-accelerating agents, which, in turn, must be harmless to humans and biologically degradable. These agents form an azeotrope with water, such as acetic acid and ethanol, and are used to accelerate drying and preserve the characteristics of the product [8,9]. Some work has been conducted using ethanol as a pretreatment to optimize the drying process of pumpkin [10] and other fruits and vegetables, such as potatoes [11], apples [12], melon [13], and pineapple [14]. The authors reported higher drying rates when compared to the drying of control samples (without pretreatment). By using different substances (acetone, water, acetic acid, and ethanol), Silva et al. [15] noted that the improvement observed may have been related to the surface tension of the compounds. In this way, they introduced the concept of flow due to the Marangoni effect on food drying. This effect is caused by the surface tension gradient that is formed between two fluids, such as water and acetic acid. The mechanism is based on the fact that the naturally formed surface tension gradient will induce the liquid to flow from regions of low to regions of high surface tension. 

Another way to enhance the quality of the product is to pretreat it with acid to change its texture and inactivate enzymes. Because certain acids have chelating qualities, a product’s color may also be preserved [16]. Hiranvarachat et al. [17] investigated the effects of citric acid pretreatment in carrot drying. The optimal conditions for carrots, according to this study, were acid blanching to pH 5 and hot air drying at 70 °C, if the physical qualities (shrinkage, rehydration ability, and color) are a concern. A visual assessment showed that this situation produced carrots with superior color, which the industry finds more appealing. However, carrots should be dried at 70 °C without any pretreatment if the retention of beta-carotene is a concern. The authors also concluded that tests and comparisons should undoubtedly be carried out with lower drying temperatures. On the other hand, the application of organic acids during pretreatment is still little explored, especially concerning improving mass transfer using these acids in surface layer lignocellulosic compounds. Through the solubilization and hydrolysis of these substances, the hardening of the food surface layer that occurs during drying can be minimized, thus improving diffusion [18]. 

There is currently one publication in the literature discussing the application of process-accelerating agents, ultrasound, and pumpkin [10]. However, the authors used ethanol in the pretreatment step and the study was performed with a different pumpkin species. Regarding other fruits or vegetables, Gorgüç et al. [19] evaluated the use of ultrasound and acid treatments when drying figs. However, their investigation had as its objective the decontamination of *Bacillus cereus*, *Penicillium expansum*, and *Escherichia coli* in dried figs using ultrasound, peroxyacetic acid, sodium chloride, and various combinations (dual/triple). The ideal sanitizing parameters were identified, and the bacteria reductions for every treatment were contrasted. Lyu et al. [20] investigated how pretreatments such as ascorbic acid, CaCl_2_, ultrasound, roasting, and heating affected the flavor quality and color retention of freeze-dried carrots over the course of their storage. Throughout the 120-day storage period, color parameters and flavor quality had constant changes that were correlated with moisture content, water activity, total carotenoid content, and lipoxygenase activity. 

The combination of ultrasound and organic acids (such as citric acid or acetic acid) can be an interesting pretreatment for vegetable drying. Additionally, although Mkhize et al. [21] reported that convective drying is the most researched method, making up around 73% of all published articles about pumpkin drying, there is also a lack of information on the effects of acid pretreatments and ultrasound on the quality parameters of dried vegetables, and their potential in optimizing the drying process still requires further studies, since how these acids act as pretreatment in different processes is still unknown. Furthermore, convective drying is crucial to the industry, which makes it much more important to assess. 

The objective of this work was to investigate the effect of organic acid combined with ultrasound as a pretreatment on pumpkin convective drying kinetics, water activity, color parameters, total phenolics, total carotenoids, and ascorbic acid contents.

## 2. Materials and Methods

### 2.1. Raw Material

Pumpkins (*Cucurbita maxima Duchesne*) were obtained from the local market of São Lourenço da Mata, Pernambuco, Brazil. The raw material was selected, cleaned, peeled, and then cut into 0.5 cm thick slices using a stainless steel knife, a cutter, and a stainless steel mold measuring 2.5 × 2.5 cm. It was then subjected to processing.

### 2.2. Ultrasound and Organic Acids Pretreatment

Pumpkin samples were immersed in a 300 mL beaker containing a 2% acetic (*v*/*v*) or citric acid (*w*/*v*) solution in a ratio of 1:4, corresponding to the weight of the sample and the weight of the solution, respectively [17,22,23]. Subsequently, the samples were placed in an ultrasonic bath model USC-2850A (Unique, Indaiatuba, Brazil) with a frequency of 25 kHz (and power of 200 W) for different times (10, 20, and 30 min). These chosen parameter values were in the range reported by Xu et al. [24] for this type of food material. The water temperature of the ultrasonic bath (30 °C) was controlled, and fluctuations in this temperature were avoided by the water circulation. This step was also analyzed in terms of water loss (*WL*) and solids gain (*SG*), as described by Azoubel et al. [25]. The weight and moisture content data of each sample were used to calculate the water loss (*WL*) (Equation (1)) and solid gain (*SG*) (Equation (2)), according to the following equations:

(1) $$WL=\frac{{ww}_{o}-(tw-ws)}{w_{o}}\times 100\%$$

(2) $$SG=\frac{ws-{ws}_{o}}{w_{o}}\times 100\%$$

where *tw* is the total wet weight of the pumpkin slice at the time of the sampling, g; *ws* is the total solids weight, g; *ws_o_* is the initial weight of solids, g; *ww_o_* the initial weight of water, g; and *w_o_* the total initial weight of the sample, g. Each experimental run was performed in triplicate.

### 2.3. Drying

The pumpkin samples with and without (control) pretreatment were dried in a stainless steel fixed bed dryer at 60 °C and an air velocity of 2.0 m/s (these parameter values were in the range reported by Xu et al. [24] for fruit and vegetables). The drying system was previously described by Medeiros et al. [26] (“The dryer system consisted of vertical airflow through trays and was arranged as a closed circuit. To maintain constant air condition only one tray was used with a single layer of sample on it. For the air heating, three electric resistances were used (two of 1600 W and one of 800 W), which could work independently, controlled by a digital thermostat. The air humidity was not monitored but the air velocity was monitored using an anemometer (Airflow, model LCS 6000, UK)”). For each experiment, nine pumpkin samples were placed in the drying tray (about 125 g each batch). Samples were weighed every 15 min until a constant weight (dynamic equilibrium) was reached.

For the drying kinetics study, five theoretical mathematical models [27] were used to adjust the experimental data (Table 1). The moisture ratio (MR) can be calculated using Equation (3).

(3) $$\frac{\bar{X}-X_{e}}{X_{0}-X_{e}}$$

where $\bar{X}$ is the average moisture content at time t, kg H_2_O∙kg^−1^ dry matter; *X_e_* is the equilibrium moisture content, kg H_2_O∙kg^−1^ dry matter; and *X*_0_ is the initial moisture content, kg H_2_O∙kg^−1^ dry matter.

As a way of analyzing the fit of the mathematical models to the experimental data, the average relative error (*P*) was calculated, which is considered acceptable when it is less than 10% [28].

(4) $$P=\frac{1}{N}\sum \frac{M_{P}-M_{0}}{M_{p}}\times 100\%$$

where *M_P_* is the expected value, *M*_0_ is the observed value, and *N* is the number of points considered in the curve.

The calculation of the effective water diffusivity (*Def*) was carried out using Equation (5) and the Statistic^®^ 10.0 Software. The effective diffusivities of water were calculated using the dimensionless moisture ratio (Equation (3)) for the first 11 terms of the series.

(5) $$MR=\frac{8}{\pi^{2}}\sum_{i=0}^{\infty }\frac{1}{{\left(2i+1\right)}^{2}}exp[-({2i+1)}^{2}\pi^{2}Def\;\frac{t}{{4l}^{2}}]$$

where *t* is time (s), i is the number of terms in the series, *Def* is the effective diffusivity of the water (m^2^ s^−1^), and *L* is the half-thickness of the food sample (m).

### 2.4. Quality Evaluation

Pumpkin (fresh and dried) samples were submitted to the quality analyses described as follows, all of which were performed in triplicate, except for color measurements, which were taken in quintuplicate (five samples from each batch). In an oven, the samples’ moisture content was determined at 105 °C for 24 h [29]. A portable analyzer model Pawkit (Decagon, Pullman, WA, USA) was used to measure the water activity (a_w_) at 25 °C.

The determination of the total carotenoid content was according to the methodology proposed by Rodriguez-Amaya [30], and the results were expressed in µg/g dry matter. The total phenolic content was measured based on the Folin–Ciocalteu reagent, as described by Singleton et al. [31]. The results were given in mg gallic acid equivalents (GAE)/g dry matter. The ascorbic acid content was quantified according to AOAC [29] and the results were given in mg of ascorbic acid/100 g of dry matter. The dry matter of the samples was obtained after determining the amount of moisture gravimetrically following the official AOAC method [29].

The color of the samples was analyzed using a calibrated portable colorimeter model CM 600D (Konica Minolta, Tokyo, Japan). The reflectance instruments determined three color parameters in the sample surface center: luminosity (L*); red (+a*) to green (−a*); and yellow (+b*) to blue (−b*). The color difference (Δ*E*) was calculated according to Medeiros et al. [26].

### 2.5. Statistical Analysis

To check whether there were significant variations between the samples, the data were subjected to analysis of variance (ANOVA). Using the Statistic^®^ 10.0 Software package, means were compared using the Tukey test at the 95% confidence level (*p* < 0.05). Each trial is presented as mean ± standard deviation (SD) and expressed as the mean of three replicates (triplicate).

## 3. Results and Discussion

### 3.1. Pretreatment

Table 2 presents the results of water loss (*WL*) and solids gain (*SG*) after the pumpkin pretreatment with ultrasound and organic acids. It can be seen that increasing the ultrasound time increased the water gain (negative water loss) and solids loss (negative solids gain) for both acids studied. Negative values of *WL* and *SG* were also found in the literature. As an example, Garcia-Noguera et al. [32] studied the effect of ultrasound exposure time as pretreatment on the osmotic dehydration of strawberries by varying the frequency and time. For the 25 kHz frequency, the researchers obtained a variation in water loss (%) from −2.7 to −3.9 and a variation in solids gain (%) from −0.1 to −0.7. Silva et al. [33], who analyzed water loss and solids gain for melon using a frequency of 25 kHz, reported values from −1.19 to −2.65% for water loss and from −1.61 to −2.30% for solids gain.

Ultrasound is responsible for the formation of microchannels in samples, also called the “sponge effect”, and these in turn favor diffusion, so the longer exposure time to ultrasound may amplify this effect [34]. Samples pretreated with acetic acid showed greater water gain and greater loss of solids than samples pretreated with citric acid, probably because acetic acid helps break down the lignocellulosic structure of the samples, which ends up facilitating mass transfer [35].

### 3.2. Drying

The pumpkins had an initial moisture content of 93.69 ± 0.07 kg water/100 kg sample (wet basis) or 14.85 ± 0.17 kg water/kg dry matter (dry basis). The results showed a high moisture content of the fresh (natura) pulp, a value similar to that provided by TACO [36] of 95.9 g of water/100 g of vegetable and to the result found by Gomes et al. [37] of 93.21 g of water/100 g of vegetable. The pumpkin samples’ moisture contents after the pretreatment with ultrasound and citric acid (AC) or acetic acid (AA) for 10, 20, and 30 min are shown in Table 3.

**Figure 1:**
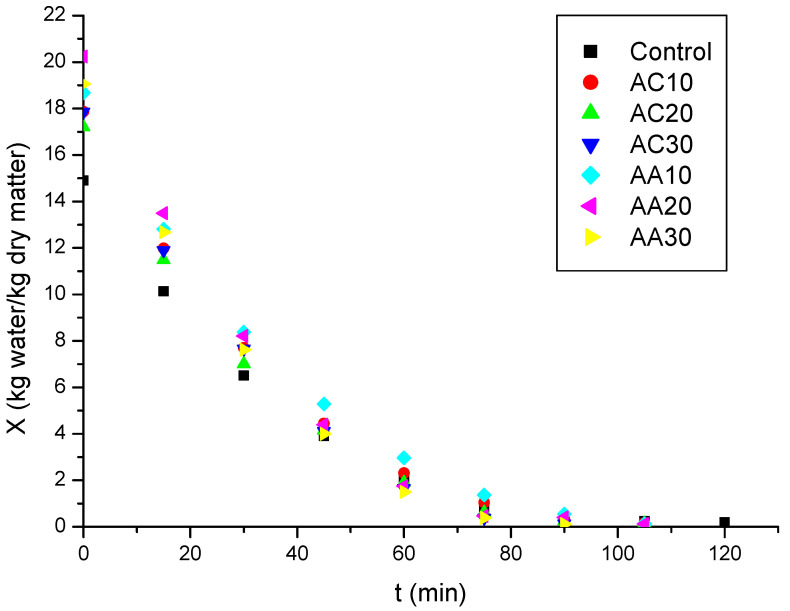
Pumpkin moisture content on a dry basis (X) as a function of drying time for the 7 conditions studied: control (without pretreatment), pretreated with citric acid (AC) or acetic acid (AA) for 10 min (AC10/AA10), 20 min (AC20/AA20), and 30 min (AC30/AA30).

**Figure 2:**
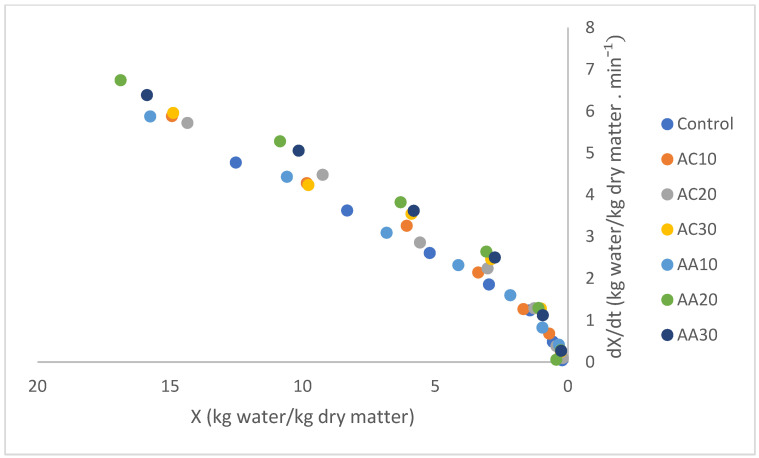
Pumpkin drying rates versus moisture content on a dry basis (X) for the 7 conditions studied: control (without pretreatment); pretreated with citric acid (AC) or acetic acid (AA) for 10 min (AC10/AA10), 20 min (AC20/AA20), and 30 min (AC30/AA30).

The study of drying kinetics and its empirical modeling is fundamental for the execution, simulation, and optimization of the process [33]. Figure 1 shows the pumpkin drying kinetic curves for all studied conditions. It was found that the control sample (without pretreatment) was the one that took the longest time to reach constant weight, that is, to reach the equilibrium condition. Villamiel et al. [38] reported that, in conventional drying, plant tissue presents turgid cells with organized and well-defined cell walls, which in turn ends up hindering water diffusivity. With pretreatment using citric acid or acetic acid solutions combined with ultrasound for 10, 20, and 30 min, it was possible to reduce the time necessary for the samples to reach dynamic equilibrium (constant weight) from 12.5% to 25.0% when comparing to the untreated dried sample (Table 4). In addition to the formation of microchannels in samples, as reported before, the use of organic acids in the pretreatment increases the permeability of pumpkin cells, which ends up increasing water diffusivity, thus facilitating drying kinetics [39,40]. From Figure 2, it can be seen that there was no constant rate period.

**Figure 3:**
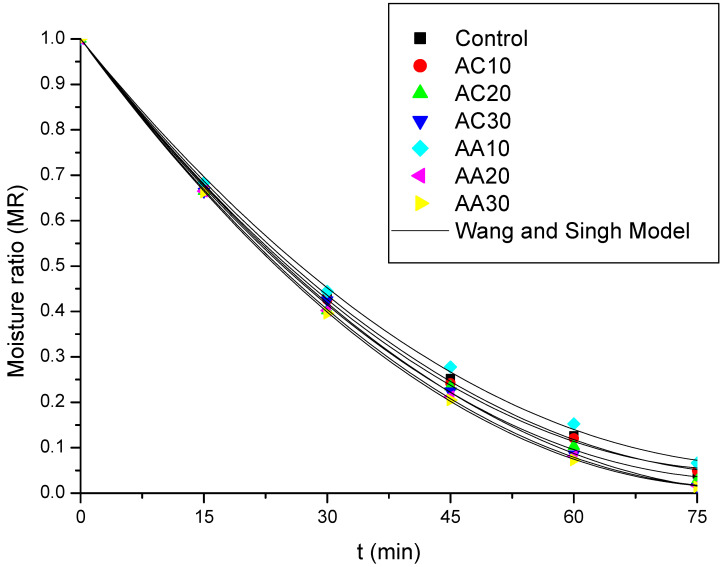
Predictive and experimental moisture ratio curves for pumpkin using the Wang and Singh model for the 7 conditions studied: control (without pretreatment), pretreated with acetic acid (AA) for 10 min (AC10/AA10), 20 min (AC20/AA20), and 30 min (AC30/AA30).

To better describe the drying kinetics behavior, empirical models are used, which make it possible to estimate the optimal processing conditions that result in the desired final moisture content. Five thin-layer kinetic models were chosen to fit the experimental data, and the models’ parameters, coefficient of determination values (R^2^), and average relative errors (*P*) are shown in Table 5. It can be seen that the Wang and Singh model is the one that presents the best fit to the experimental data (Figure 3), with the highest values of R^2^ and the lowest values of *P* (%), which are also less than 10% (Table 5).

The Wang and Singh equation was the one that demonstrated the greatest effectiveness in modeling experimental data, and several authors reported good adjustments using this equation. Doymaz [41], Koç and Dirim [42], and Oliveira et al. [43] observed that the Wang and Singh equation presented a good fit to the kinetic drying data of pumpkin, pumpkin puree, and pumpkin seeds, respectively. Wang and Singh’s model was also the one that best adjusted the kinetic curves evaluated by Mahapatra and Tripathy [44], Smaniotto et al. [45], and Khawas et al. [46], in which solar drying of carrots, sunflower seeds, and bananas, respectively, were studied.

It was observed that the pumpkin drying presented the highest value for the Wang and Singh model, a parameter for the AA30 sample (Table 5), which resulted in a lower final moisture content and a shorter drying time when compared to the other conditions studied. Similar behavior was observed by Mahapatra and Tripathy [44], and Khawas et al. [46], in the same way, noticed an increase in *a* parameter value with an increase in the drying rate when studying banana drying at 40, 50, 60, and 70 °C. Smaniotto et al. [45] also observed this behavior with increasing temperatures (35, 50, 65, 80, and 95 °C) during the drying of sunflower seeds.

Acetic acid pretreatment, when compared to citric acid pretreatment, managed to remove a greater water content when the drying times were identical. This behavior can be seen in Table 5, where the parameter *a* of AA30 drying is greater than that of AC30 drying, indicating greater water diffusivity in the pretreatment with acetic acid. A possible explanation for this is the fact that acetic acid is used in the wood and cellulose industry as a way to break down lignocellulosic structures and, thus, obtain greater access to cellulose [35]. During pumpkin drying, a dry layer forms on the surface of the food, the formation of which ends up making mass transfer difficult. So, acetic acid may be breaking down this dry layer and, in turn, making it easier to remove water from the product.

Citric acid, when analyzed from the point of view of the acidity constant (Ka), is a stronger acid than acetic acid, as they have Ka values of 8 × 10^−4^ and 1.8 × 10^−5^, respectively [47]. It is not necessarily true that a stronger organic acid will be more efficient in removing water during drying when used as a pretreatment. For example, Dufera et al. [48] observed that pretreatment with 0.5% (*w*/*v*) ascorbic acid (Ka = 8 × 10^−5^, as reported by Reger et al. [49]) was more efficient in terms of reducing moisture content than pretreatment with 0.5% (*w*/*v*) citric acid (Ka = 8 × 10^−4^) when they studied tomato drying in a solar tunnel. They reported that after 4 h of drying, the samples pretreated with ascorbic acid showed a moisture content on a wet basis of 20.57%, while samples pretreated with citric acid during the same drying time showed a final moisture content of 29.5%. In Table 4, information can be found on the final moisture contents of each of the conditions studied for pumpkins.

The obtained effective moisture diffusivities (Def) of the samples are shown in Table 6. Generally, for foods, the diffusivity coefficient is between 10^−12^ and 10^−8^ m^2^∙s^−1^ [50]. Onwude et al. [51], studying the drying kinetics of pumpkin at different temperatures and thicknesses, found that for a drying condition at 60 °C and a thickness of 5 mm, the diffusivity values found were in the order of 10^−8^, as in our study.

The pumpkin samples submitted to the pretreatment step presented higher diffusivity values (except in the case of the AA10 condition) when compared to the control sample, with the AA20 and AA30 pretreatments being the ones that presented the highest diffusivity values. The use of citric acid or acetic acid associated with ultrasound facilitated the removal of water from the interior to the surface of the sample, thus increasing the diffusivity by 9.43%, going from 6.68 × 10^−8^ m^2^∙s^−1^ to 7.31 × 10^−8^ m^2^∙s^−1^. Doymaz [41], studying the convective drying of a different pumpkin variety (*Cucurbita pepo* L.) at 60 °C, also reported that the pretreatment was efficient in increasing the effective diffusivity of the vegetable.

### 3.3. Quality Analysis

Brazilian legislation determines, for dried vegetables, a maximum moisture content value of 12 kg water/100 kg sample (wet basis) [52]. Therefore, the quality analyses were performed for pumpkin-dried samples that reached this moisture content, and only the AC30, AA10, AA20, and AA30 dried samples continued for quality analysis. The dried samples without pretreatment and AC10 and AC20 samples did not reach the moisture content and were excluded from quality analyses, which is discussed as follows.

#### 3.3.1. Water Activity (a_w_)

Water activity plays a relevant role in different biological processes, especially in the development of microorganisms, as it is a measure of the free water that is present in a biological material. Thus, it is possible to estimate the intensity of the association of water with non-aqueous components [53]. The water activity (a_w_) values of the fresh and dried pumpkin samples are shown in Table 7.

The fresh pumpkin had a water activity of 0.99, a value close to that found by Köprüalan et al. [54], which was 0.98. The combined use of ultrasound with citric acid or acetic acid, followed by drying, managed to significantly reduce the water activity, with acetic acid being a little bit more efficient than citric acid in reducing this parameter. A similar behavior was observed by Dufera et al. [48], who studied the drying of tomatoes in a solar tunnel. It was observed that the water activity of fresh tomatoes was 0.95, and, after 6 h of drying, the tomatoes pretreated with a 0.5% citric acid solution (*w*/*v*) had their water activity reduced to 0.336. According to Islam et al. [55], in general, microorganisms will not grow in food products whose water activity is less than 0.62. This shows that pretreatment in conjunction with drying can allow for a longer shelf life.

#### 3.3.2. Total Phenolics, Total Carotenoids, and Ascorbic Acid Contents

A common preservation method for fruits and vegetables is heat processing, which lowers the microbial and enzyme activity in the produce and prolongs its shelf life [56]. However, the use of high temperatures had negative effects on the fruit and vegetable quality attributes, primarily because it sped up the fruit and vegetable metabolism of nutritional compounds like ascorbic acid, carotenoids, and polyphenols [57].

Preserving the phenolic content during drying is essential, since phenolic compounds, found in plants, are extremely relevant to the human diet and attract attention due to their antioxidant qualities [58]. The levels of phenolic compounds in fresh and dried pumpkin are shown in Table 7. The phenolic content for the fresh samples was 0.64 mg GAE/g dry matter, a value different from those found by Mohammed [59], who reported a value of 3.15 mg GAE/g in solar drying of pumpkin pulp powder. The differences found in the phenolic content could be related to the composition of the pumpkins, which, in turn, is influenced by factors such as variety, irrigation technique, harvest time, place where it was produced, climate, and type of soil.

After drying, it can be seen that there was a reduction in the phenolic content of the pumpkin samples. This is because phenolic compounds are sensitive to heat and oxygen [60]. Enzymatic oxidation caused by polyphenol oxidase was described by Djendoubi [61] as the main mechanism by which phenol degradation occurs during convective drying. Also, Fonteles et al. [62] observed a decrease of up to 30% in the total phenolic content in melon juice samples subjected to ultrasound. The researchers noted that there was formation of free radicals, and this may have impacted the phenolics in the melon juice, as the -OH radicals formed during ultrasonic cavitation can affect bioactive compounds such as phenolics.

The best retention of phenolic compounds was in the AC30 and AA10 samples. In the first case, citric acid is less aggressive to cellular tissue than acetic acid, which allows for greater retention of water-soluble compounds such as phenolics. Also, according to Dyab et al. [63], citric acid facilitated the decrease in the oxidation rate by the enzymes polyphenol oxidase and peroxidase. In food processing, citric acid is used to enhance texture and inhibit browning.

Carotenoids are lipophilic organic compounds with known antioxidant capacity. Some carotenoids have provitamin A activity, such as β-carotene, which is important in controlling cardiovascular diseases [64]. The carotenoid content found for fresh samples was 91.93 μg/g dry matter (Table 7), a value within the range found by Kreck et al. [65] when studying different varieties of pumpkin, which ranged from 17 μg/g to 683 μg/g dry matter.

There was a reduction in the carotenoid content after drying (Table 7), which was already expected, as carotenoids are compounds sensitive to the action of several factors such as light, heat, enzymes, and oxygen [14]. However, there was a greater retention of carotenoids in AA10 and AA20 samples, which have a longer drying time compared to the samples AC30 and AA30. This can be explained by the fact that these latter samples were exposed to oxygen for a longer time during pretreatment with ultrasound, which favored a decrease in the carotenoid content. Rodrigues-Amaya [66] reported that oxygen reacts with carotenoids producing free radicals, since the mechanism of action of carotenoids is to chelate oxygen. Also, as verified by Hiranvarachat et al. [17], following the pretreatment of carrots, there was a marked decline in the retention of β-carotene. As β-carotene is unstable in acidic environments, there was a decrease in its retention [67]. Moreover, acid may react with β-carotene by protonating either a β-carotene molecule or a β-carotene double bond [68]. Lower retention of β-carotene in the case of the carrot resulted from contact with acid because hydrocarbons like β-carotene might be oxidized by it and produce a new molecule. On the other hand, in the case of acid soaking, the retention of β-carotene remained nearly constant during carrot drying. These outcomes corroborated those of Veda et al. [69], who suggested that acidulants such as citric acid stopped β-carotene from being lost during heat processing.

Regarding ascorbic acid content, samples of fresh pumpkins showed levels of 128.71 mg/100 g dry matter (Table 7). Similar values were reported by Ouyang et al. [70], who found a value of 110.29 mg/100 g dry matter, and by Gonçalves et al. [71], who reported 113.67 mg/100 g dry matter. Ascorbic acid content was considerably reduced after drying. This was because it is very sensitive to heat, oxygen, and light and is water-soluble, being extracted from pumpkin into the organic acid solution, which ends up being enhanced due to the action of ultrasound, as reported by Arruda et al. [72] for papaya. An important factor to be evaluated is that during pretreatment, excess free radicals can be formed through sonochemical reactions, thus enhancing oxidative processes [34].

Ouyang et al. [70] reported a retention of 51.0% for pumpkin samples dried at 60 °C for 17 h, a value slightly higher than that found for AC30 samples. Even though ultrasonic pretreatment is, in general, detrimental to the retention of ascorbic acid, it is possible to explain why the results are similar, as the shorter drying time for the AC30 samples (90 min) ended up reducing the exposure time of the samples to heat and oxygen, which helped to preserve ascorbic acid. The samples pretreated with citric acid (AC30) were those that showed the greatest retention of ascorbic acid. This can be explained by the fact that acetic acid is more aggressive to the pumpkin’s cellular tissue, which ended up making it less rigid, facilitating the extraction of ascorbic acid from the interior of the cells to the external environment. The longer exposure time to ultrasound ended up increasing the loss of ascorbic acid from samples pretreated with acetic acid. Carvalho et al. [73], comparing the action of four substances (ethanol, acetone, isopropanol, and acetic acid) on pumpkin drying, observed that the samples that were pretreated with 99% acetic acid (*v*/*v*) showed greater structural changes in the internal tissue cells.

#### 3.3.3. Color

Color is an important attribute for evaluating the quality and appearance of food and also influencing consumer preferences [74]. The results of the color evaluation of pumpkins are shown in Table 7. It can be seen that the pretreated samples did not significantly differ much from each other in the color attributes evaluated (95% confidence level), given that the greatest noticeable difference was between the fresh and the dried pretreated samples.

There was a slight increase in the luminosity value (L*) of the dried samples when compared to the fresh ones. The use of citric and acetic acids probably protected the samples from enzymatic browning in some way by inhibiting the action of enzymes, such as peroxidase and polyphenol oxidase, responsible for the browning of vegetables, thus resulting in luminosity values very close to the value of fresh samples. It is thought to be the influence of pH on Maillard reactions, wherein an alkaline pH favors the reaction’s initial step, which is pH-dependent [75,76]. Because the amine groups are largely protonated and thus unavailable for the reaction, an acid pH or pH lower than the pK of the amine groups reduces the reaction [77]. Similar results were found by Doymaz [78] based on his studies on drying kiwifruit at 50 °C, 55 °C, 60 °C, and 70 °C using a 1% citric acid solution as pretreatment, and by Sun et al. [79] when analyzing the drying of potatoes at 50 °C, 60 °C, and 70 °C using citric acid solutions of concentrations of 0.1%, 0.2%, and 0.3% as pretreatment.

There was a slight decrease in a* values when comparing the dried pretreated with the fresh samples, which means that there was a reduction in the red color of the samples that underwent pretreatment. This may be related to ultrasound, as during ultrasonic cavitation, some pigments responsible for the pumpkin’s redder color may have been extracted from the samples. This leaching effect during sonication has also been reported as the cause of a* reduction in ultrasonic pretreated dried apples [6] and papaya [72]. Also, Onwude et al. [74] mentioned that the autoxidation of carotenoids, which gradually caused the pumpkin’s red color to change at much higher temperatures and longer drying times, may be the reason for the drop in the a* value during drying.

Concerning the b* parameter, the pretreated samples showed an increase compared to the fresh samples, which means that there was an increase in the yellow color of the samples that underwent pretreatment. Similar results were found by Dyab et al. [63] when studying the drying of potatoes at 60 °C and using a 1% citric acid solution as pretreatment. Sahoo et al. [80] observed that the rise in the b* value implied that heat could weaken the matrix of the cell wall and accelerate the release of carotenoids when drying yam slices. They also verified that between 50 and 70 °C, a light to deeper yellow and brown color characteristic was noted. The dried samples at 70 °C showed faster development in the dark brown color of the slices. This could be because of the carotenoids’ breakdown and non-enzymatic Maillard reaction, which gives the dried slices a reddish-brown color with a hint of yellow [81,82].

Color variation (Δ*E*) is used as an indicator that compares the color change between fresh samples and dried samples. The obtained Δ*E* values may be the result of color changes caused by pretreatment (leaching into the pigment solution and damage to the cell membrane) and the drying process (oxidation of carotenoids, phenolic compounds, and ascorbic acid and non-enzymatic oxidation) [83,84]. The samples presented a high value for Δ*E,* the values were very close to each other, and there were no significant differences among them. Similar behavior was reported by Doymaz [78] with the study of drying kiwifruit at 50 °C, 55 °C, 60 °C, and 70 °C pretreated with a 1% (*w*/*w*) citric acid solution, which found color variations (Δ*E*) for samples pretreated with citric acid of 8.719, 7.881, 7.226, and 7.216 at temperatures of 50 °C, 55 °C, 60 °C, and 70 °C, respectively. Onwude et al. [74] studied the drying kinetics of pumpkin and found color variations (Δ*E*) that ranged from 10.6 to 17.46 in a temperature range of 50 °C to 80 °C.

## 4. Conclusions

The results of this work demonstrate that the use of citric or acetic acid in conjunction with ultrasound as pretreatment had a positive influence on reducing the drying time of pumpkin compared to control drying (without pretreatment). The drying kinetics study showed that it was only possible to reach a 12% (on a wet basis) moisture content, as is required by Brazilian legislation for vegetables, when the pumpkin was pretreated with ultrasound and citric (AC30) or acetic (AA10, AA20, and AA30) acids. Pumpkin pretreated with acid and ultrasound for 30 min resulted in the shortest drying times, and the Wang and Singh model was the most predictive in the mathematical modeling of this process. The effective moisture diffusivity was found to be in the range of 6.24 × 10^−8^ to 7.31 × 10^−8^ m^2^∙s^−1^.

The evaluated dried samples’ water activity was less than 0.60, and the ones pretreated with citric acid and ultrasound for 30 min (AC30) had the best retention of ascorbic acid and total phenolic compounds, followed by the pumpkin treated with acetic acid and ultrasound for 10 min (AA10). However, pumpkin samples that had been processed with acetic acid and ultrasound for 10 min (AA10) and 20 min (AA20) showed higher total carotenoid retention. Compared to the fresh pumpkin samples, all of the pretreated samples had increases in lightness and yellowness and decreases in redness.

Among the studied conditions, the AA10 pretreatment of pumpkin simultaneously presented good results from a kinetic point of view, which is important to lower the processing cost and is also helpful for the retention of bioactive compounds in dried pumpkin. Thus, there is much promise for agricultural product preservation with the combination of ultrasound and organic acids. Nonetheless, further research is still required in order to study other process conditions (like ultrasound frequency, solution concentration, other acids, etc.) and to determine whether this approach is viable on a large scale and on a pilot plant level.

## Figures and Tables

**Table 1:** Thin-layer models used for pumpkin drying.

Model	Equation
Single exponential	MR=exp(−kt)
Henderson and Pabis	MR=a exp(−kt)
Logarithmic	MR=a exp(−kt)+c
Two-term exponential	MR=a exp⁡(−kt)+b exp⁡(−wt)
Wang and Singh	$MR=1+at+bt^{2}$

*k* (s^−1^), a (for Wang and Singh model: s^−1^), *b* (for Wang and Singh model: s^−2^), *c*, *b*, *w* (s^−1^): parameters in thin-layer models; *MR*: moisture ratio.

**Table 2:** Water loss (*WL*) and solids gain (*SG*) during pumpkin pretreatment with ultrasound and citric acid (AC) or acetic acid (AA) for 10 min (AC10/AA10), 20 min (AC20/AA20), and 30 min (AC30/AA30).

Sample	*WL* (%)	*SG* (%)
AC10	−0.56 ± 0.03 ^a^	−0.13 ± 0.02 ^a^
AC20	−1.19 ± 0.08 ᵇ	−0.27 ± 0.04 ᵇ
AC30	−1.49 ± 0.07 ^c^	−0.35 ± 0.03 ^c^
AA10	−1.12 ± 0.10 ᵇ	−0.25 ± 0.02 ᵇ
AA20	−1.65 ± 0.14 ^c^	−0.46 ± 0.01 ᵈ
AA30	−2.03 ± 0.15 ᵈ	−0.52 ± 0.03 ᵉ

Mean ± standard deviation (SD) with the same letter within the same column showed no statistically significant difference for their mean values at a 95% confidence level.

**Table 3:** Pumpkin moisture content (X) after pretreatment with citric acid (AC) or acetic acid (AA) for 10 min (AC10/AA10), 20 min (AC20/AA20), and 30 min (AC30/AA30).

Sample	X (Wet Basis, kg Water/100 kg Sample)	X (Dry Basis, kg Water/kg Dry Matter)
AC10	94.25 ± 0.03	16.39 ± 0.08
AC20	94.88 ± 0.08	18.53 ± 0.14
AC30	95.18 ± 0.07	19.75 ± 0.16
AA10	94.81 ± 0.10	18.27 ± 0.21
AA20	95.34 ± 0.14	20.46 ± 0.30
AA30	95.72 ± 0.15	22.36 ± 0.32

**Table 4:** Pumpkin drying time and final moisture content (dynamic equilibrium) for control (untreated), pretreated with citric acid (AC) or acetic acid (AA) for 10 min (AC10/AA10), 20 min (AC20/AA20), and 30 min (AC30/AA30).

Samples	Time to Reach Equilibrium Moisture Content (min)	Time Reduction Compared to Untreated Sample (min)	Final Moisture Content (kg Water/kg Sample)	Final Moisture Content (kg Water/kg Dry Matter)
Control	120	-	16.03 ± 0.14 ^a^	0.19 ± 0.01 ^a^
AC10	105	12.5	14.17 ± 0.13 ᵇ	0.17 ± 0.01 ᵇ
AC20	105	12.5	12.43 ± 0.12 ^c^	0.14 ± 0.01 ^c^
AC30	90	25.0	11.70 ± 0.11 ᵈ	0.13 ± 0.01 ᵈ
AA10	105	12.5	11.80 ± 0.03 ᵈ	0.13 ± 0.01 ᵈ
AA20	105	12.5	11.06 ± 0.07 ᵉ	0.12 ± 0.01 ᵉ
AA30	90	25.0	10.54 ± 0.11 ᶠ	0.12 ± 0.01 ᶠ

Mean ± standard deviation (SD) with the same letter within the same column showed no statistically significant differences for their mean values at a 95% confidence level.

**Table 5:** Pumpkin drying models’ parameters for control (without pretreatment), pretreated with citric acid (AC) or acetic acid (AA) for 10 min (AC10/AA10), 20 min (AC20/AA20), and 30 min (AC30/AA30).

Models	Sample	Parameters				R^2^	*P* (%)
Henderson and Pabis		*a*	*k*				
	Control	1.0232	0.0312			0.9901	28.0948
	AC10	1.0222	0.0317			0.9909	25.7271
	AC20	1.0246	0.0331			0.9888	44.0220
	AC30	1.0286	0.0335			0.9827	95.6486
	AA10	1.0194	0.0294			0.9932	16.0379
	AA20	1.0291	0.0343			0.9847	75.4536
	AA30	1.0300	0.0349			0.9839	89.3456
Logarithmic		*a*	*k*	*c*			
	Control	1.2009	0.0216	−0.1988		0.9999	1.9995
	AC10	1.1822	0.0225	−0.1797		0.9998	3.7916
	AC20	1.1952	0.0230	−0.1917		0.9997	5.0739
	AC30	1.2604	0.0210	−0.2577		0.9993	17.1681
	AA10	1.1726	0.0213	−0.1714		0.9999	0.8006
	AA20	1.1772	0.0212	−0.1761		0.9999	0.8608
	AA30	1.2246	0.0230	−0.2181		0.9989	21.0980
Two-term exponential		*a*	*k*	*b*	*w*		
	Control	0.5179	0.0312	0.5053	0.0312	0.9901	28.0950
	AC10	0.5501	0.0317	0.4721	0.0317	0.9909	25.7276
	AC20	−42.9453	0.0283	43.9469	0.0284	0.9992	9.2724
	AC30	−73.2640	0.0281	74.2643	0.0283	0.9982	25.5953
	AA10	−95.6213	0.0248	96.6198	0.0250	0.9996	2.2760
	AA20	−61.0873	0.0297	62.0896	0.0230	0.9987	21.2206
	AA30	−84.5977	0.0304	85.6013	0.0303	0.9985	27.0797
Wang and Singh		*a*	*b*				
	Control	−0.0229	0.000137			0.9994	5.4474
	AC10	−0.0234	0.000143			0.9995	4.4087
	AC20	−0.0239	0.000148			0.9992	7.5585
	AC30	−0.0236	0.000140			0.9997	2.3111
	AA10	−0.0222	0.000131			0.9991	4.5355
	AA20	−0.0243	0.000150			0.9999	2.6132
	AA30	−0.0247	0.000154			0.9999	1.3180
Single exponential		*k*					
	Control	0.0305				0.9891	29.3033
	AC10	0.0311				0.9900	27.0462
	AC20	0.0324				0.9878	45.7841
	AC30	0.0326				0.9813	101.6866
	AA10	0.0288				0.9925	16.7395
	AA20	0.0334				0.9834	78.9527
	AA30	0.0340				0.9825	93.5021

**Table 6:** Pumpkin effective diffusivities (D_ef_) for control (untreated), pretreated with citric acid (AC) or acetic acid (AA) for 10 min (AC10/AA10), 20 min (AC20/AA20), and 30 min (AC30/AA30) submitted to convective drying.

Sample	D_ef_ × 10^8^ (m^2^∙s^−1^)	R^2^
Control	6.68 ± 0.15 ᵇ	0.9658
AC10	6.73 ± 0.15 ᶜ	0.9655
AC20	7.02 ± 0.04 ᵈ	0.9646
AC30	7.01 ± 0.13 ᵈ	0.9549
AA10	6.24 ± 0.05 ᵃ	0.9655
AA20	7.22 ± 0.10 ᵉ	0.9615
AA30	7.31 ± 0.06 ᶠ	0.9570

Mean ± standard deviation (SD) with the same letter within the same column showed no statistically significant differences in their mean values at a 95% confidence level.

**Table 7:** Quality analyses of pumpkin: fresh and dried pretreated with citric acid (AC) or acetic acid (AA).

Methods	Water Activity (a_w_)	Total Phenolics Content (mg GAE∙100 g^−1^ DM)	Total Carotenoids Content (µg∙g^−1^ DM)	Ascorbic Acid Content (mg/∙100 g^−1^ DM)	Color			
					L*	a*	b*	Δ*E*
Fresh	0.99 ± 0.01 ᵃ	0.64 ± 0.02 ^a^	91.93 ± 1.82 ^a^	128.71 ± 8.17 ᵃ	77.70 ± 0.03 ᵇ	17.12 ± 1.28 ᵃ	44.07 ± 1.42 ᵇ	-
AC30	0.59 ± 0.02 ᵇ	0.58 ± 0.01 ᵇ	30.52 ± 0.23 ᵈ	63.39 ± 1.54 ᵇ	79.61 ± 1.16 ᵃ	15.37 ± 2.35 ᵃᵇ	63.93 ± 2.26 ᵃ	18.17 ± 0.72 ᵃ
AA10	0.51 ± 0.01 ᶜ	0.58 ± 0.01 ᵇ	50.47 ± 0.29 ᵇ	42.39 ± 0.91 ^c^	78.94 ± 0.23 ᵃ	13.08 ± 0.34 ᵇ	61.80 ± 1.25 ᵃ	18.51 ± 0.56 ᵃ
AA20	0.51 ± 0.03 ᶜ	0.52 ± 0.03 ^c^	51.10 ± 1.12 ᵇ	31.48 ± 0.90 ᵈ	79.29 ± 0.50 ᵃ	14.11 ± 0.08 ᵇ	63.79 ± 0.67 ᵃ	18.90 ± 2.53 ᵃ
AA30	0.51 ± 0.03 ᶜ	0.45 ± 0.01 ᵈ	45.71 ± 0.69 ͨ	20.93 ± 0.49 ᵉ	80.10 ± 0.40 ᵃ	13.99 ± 0.01 ᵇ	62.53 ± 0.15 ᵃ	17.00 ± 1.00 ᵃ

Means ± standard deviations (SDs) with the same letter within the same column showed no statistically significant differences for their mean values at 95% confidence level. DM: dry matter.

## Data Availability

The original contributions presented in the study are included in the article, further inquiries can be directed to the corresponding author.

## References

[1] Ropelewska E., Popinska W., Sabanci K., Aslan M.F. (2022). Flesh of pumpkin from ecological farming as part of fruit suitable for non-destructive cultivar classification using computer vision. Eur. Food Res. Technol..

[2] Kulczynski B., Gramza-Michałowska A. (2019). The profile of secondary metabolites and other bioactive compounds in *Cucurbita pepo* L. and *Cucurbita moschata* pumpkin cultivars. Molecules.

[3] Chikpah S.K., Korese J.K., Sturm B., Hensel O. (2022). Colour change kinetics of pumpkin (*Cucurbita moschata*) slices during convective air drying and bioactive compounds of the dried products. J. Agric. Food Res..

[4] Zubernik J., Dadan M., Cichowska J., Witrowa-Rajchert D. (2020). The impact of the pre-treatment in ethanol solution on the drying kinetics and selected properties of convective dried apples. Int. J. Food Eng..

[5] Huang D., Men K., Li D., Wen T., Gong Z., Sundenzan B., Wu Z. (2020). Application of ultrasound technology in the drying of food products. Ultrason. Sonochem..

[6] Nowacka M., Wiktor A., Śledź M., Jurek N., Witrowa-Rajchert D. (2012). Drying of ultrasound pretreated apple and its selected physical properties. J. Food Eng..

[7] Miano A.C., Rojas M.L., Augusto P.E.D. (2017). Other mass transfer unit operations enhanced by ultrasound. Ultrasound: Advances in Food Processing and Preservation.

[8] Braga A.M.P., Pedroso M.P., Augusto F., Silva M.A. (2009). Volatiles identification in pineapple submitted to drying in an ethanolic atmosphere. Dry. Technol..

[9] Braga A.M.P., Silva M.A. Effect of ethanol on the drying kinetics and on the quality of pineapple slices. Proceedings of the 17th International Drying Symposium.

[10] Rojas M.L., Silveira I., Augusto P.E.D. (2020). Ultrasound and ethanol pre-treatments to improve convective drying: Drying, rehydration and carotenoid content of pumpkin. Food Bioprod. Process..

[11] Rojas M.L., Augusto P.E.D. (2018). Ethanol and ultrasound pre-treatment to improve infrared drying of potato slices. Innov. Food Sci. Emerg. Technol..

[12] Rojas M.L., Augusto P.E.D., Cárcel J.A. (2020). Ethanol pre-treatment to ultrasound-assisted convective drying of apple. Innov. Food Sci. Emerg. Technol..

[13] Cunha R.M.C., Brandão S.C.R., Medeiros R.A.B., Silva Júnior E.V., Silva J.H.F., Azoubel P.M. (2020). Effect of ethanol pretreatment on melon convective drying. Food Chem..

[14] de Freitas L.D.C., Brandão S.C.R., da Silva J.H.F., da Rocha O.R.S., Azoubel P.M. (2021). Effect of ethanol and ultrasound pretreatments on pineapple convective drying. Food Technol. Biotech..

[15] Silva M.A., Braga A.M.P., Santos P.H.S. Enhancement of fruit drying: The ethanol effect. Proceedings of the 18th International Drying Symposium (IDS 2012).

[16] Gallmann P. (2002). Food Additives: Second edition, revised and expanded. A.L. Branen, P.M. Davidson, S. Salminen, J.H. Thorngate; Marcel Dekker, New York, Basel, 2002. Int. Dairy J..

[17] Hiranvarachat B., Devahastin S., Chiewchan N. (2011). Effects of acid pretreatments on some physicochemical properties of carrot undergoing hot air drying. Food Bioprod. Process..

[18] Tomás-Pejó E., Alvira P., Ballesteros M., Negro M.J., Larroche C., Ricke S.C., Dussap C.-G., Gnansounou E. (2011). Chapter 7—Pretreatment Technologies for Lignocellulose-to-Bioethanol Conversion.

[19] Gorgüç A., Gençdağ E., Okuroğlu F., Yılmaz F.M., Halil Bıyık H., Kose Köse S.Ö., Ersus S. (2021). Single and combined decontamination effects of power-ultrasound, peroxyacetic acid and sodium chloride sanitizing treatments on *Escherichia coli*, *Bacillus cereus* and *Penicillium expansum* inoculated dried figs. LWT.

[20] Lyu Y., Bi J., Chen Q., Li X., Wu X., Gou M. (2022). Effects of ultrasound, heat, ascorbic acid and CaCl_2_ treatments on color enhancement and flavor changes of freeze-dried carrots during the storage period. Food Chem..

[21] Mkhize X., Nkosi N., Zondi L., Tumba K. (2023). Convective drying of pumpkin: Brief literature review and new data for organically produced indigenous pumpkin (*Cucurbita pepo* L.) over an expanded temperature range. J. Agric. Food Res..

[22] Chiewchan N., Praphraiphetch C., Devahastin S. (2010). Effect of pretreatment on surface topographical features of vegetables during drying. J. Food Eng..

[23] Zhao W., Shehzad H., Yan S., Li J., Wang Q. (2017). Acetic acid pretreatment improves the hardness of cooked potato slices. Food Chem..

[24] Xu B., Tiliwa E.S., Yan W., Roknul Azam S.M., Wei B., Zhou C., Ma H., Bhandari B. (2022). Recent development in high quality drying of fruits and vegetables assisted by ultrasound: A review. Food Res. Int..

[25] Azoubel P.M., Baima M.A.M., Amorim M.R., Oliveira S.S.B. (2010). Effect of ultrasound on banana cv Pacovan drying kinetics. J. Food Eng..

[26] Medeiros R.A.B., Barros Z.M.P., Carvalho C.B.O., Neta E.G.F., Maciel M.I.S., Azoubel P.M. (2016). Influence of dual-stage sugar substitution pretreatment on drying kinetics and quality parameters of mango. LWT.

[27] da Silva E.S., Brandão S.C.R., da Silva A.L., da Silva J.H.F., Coêlho A.C.D., Azoubel P.M. (2019). Ultrasound-assisted vacuum drying of nectarine. J. Food Eng..

[28] Lomauro C.J., Bakshi A.S., Labuza T.P. (1985). Evaluation of food moisture sorption isotherm equations. Part I: Fruit, vegetable and meat products. LWT.

[29] AOAC (2002). Official Methods of Analysis of the Association of Official Analytical Chemists.

[30] Rodriguez-Amaya D.B. (1999). A Guide to Carotenoid Analysis in Foods.

[31] Singleton V.L., Orthofer R., Lamuela R.M. (1999). Analysis of total phenols and other oxidation substrates and antioxidants by means of Folin Ciocalteau reagent. Methods Enzymol..

[32] Garcia-Noguera J., Oliveira F.I.P., Gallão M.I., Weller C., Rodrigues S., Fernandes F.A.N. (2010). Effect of ultrasonic and osmotic dehydration pre-treatments on the colour of freeze dried stawberries. J. Food Sci. Technol..

[33] Silva J.H.F., Galvão C.C., Silva E.S., Cavalcanti D.E.S., Rocha O.R.S., Azoubel P.M., Benachour M. Secagem convectiva de melão (*Cucumis melo* L.) com e sem pré-tratamento ultrassônico. Proceedings of the XXII Congresso Brasileiro de Engenharia Química.

[34] Wang J., Wang J., Ye J., Kranthi S., Raghavan V. (2019). Influence of high-intensity ultrasound on bioactive compounds of strawberry juice: Profiles of ascorbic acid, phenolics, antioxidant activity and microstructure. Food Control.

[35] Padilha C.E.A., Nogueira C.C., Oliveira Filho M.A., Souza D.F.S., Oliveira J.A., Santos E.S. (2020). Valorization of cashew apple bagasse using acetic acid pretreatment: Production of cellulosic ethanol and lignin for their use as sunscreen ingredients. Process Biochem..

[36] TACO (2011). Tabela Brasileira de Composição de Alimentos.

[37] Gomes E.S., Marins A.R., Gomes R.G. (2022). Avaliação das características químicas e físicas da farinha de abóbora (Cucurbita maxima): Polpa e sementes. Res. Soc. Dev..

[38] Villamiel M., Garcia-Perez J.V., Montilla A., Cárcel J.A., Benedito J. (2017). Ultrasound in Food Processing: Recent Advances.

[39] Ozdemir Y., Ozturk A., Tüfekçi S. (2016). Effect of two dipping pretreatment on drying kinetics of golden berry (*Physalis peruviana* L.). Afr. J. Agric. Res..

[40] Osidacz R.C., Anbrosio-Ugri M.C.B. (2013). Physicochemical quality of eggplant dehydrated with varied pretreatments. Acta Sci. Technol..

[41] Doymaz I. (2007). The kinetics of forced convective air-drying of pumpkin slices. J. Food Eng..

[42] Koç G.Ç., Dirim N. Determination of the freeze drying kinetics of pumpkin (*Cucurbita moschata*) Puree. Proceedings of the 19th International Drying Symposium (IDS2014).

[43] Oliveira D.E.C., Guimarães J.M., Bueno J.M.G.S., Costa Júnior J.R., Alves E.M., Silva B.M.C. (2021). Drying kinetics of pumpkin seeds. Comun. Sci..

[44] Mahapatra A., Tripathy P.P. (2018). Modeling and simulation of moisture transfer during solar drying of carrot slices. J. Food Process Eng..

[45] Smaniotto T., Resende O., Sousa K., Oliveira D., Campos R. (2017). Drying kinetics of sunflower grains. Rev. Bras. Eng. Agric. Ambient..

[46] Khawas P., Dash K., Das A., Deka S. (2015). Drying characteristics and assessment of physicochemical and microstructural properties of dried culinary banana slices. Int. J. Food Eng..

[47] Skoog D., West D., Holler J., Crouch S. (2006). Fundamentos de Química Analítica.

[48] Dufera L.T., Hofacker W., Esper A., Hensel O. (2022). Effect of different predrying treatments on physicochemical quality and drying kinetics of twin layer solar tunnel dried tomato (*Lycopersicon esculentum* L.) slices. J. Food Qual..

[49] Reger D., Goode S., Mercer E. (2010). Química: Princípios e Aplicações.

[50] Jha A.K., Sit N. (2020). Drying characteristics and kinetics of colour change and degradation of phytocomponents and antioxidant activity during convective drying of deseeded *Terminalia chebula* fruit. J. Food Meas. Charact..

[51] Onwude D., Hashim N., Janius R., Nawi N., Abdan K. (2013). Modelling effective moisture diffusivity of pumpkin (*Cucurbita moschata*) slices under convective hot air drying condition. Int. J. Food Eng..

[52] BRASIL (2005). Ministério da Saúde. Resolução—RDC n° 272, de 22 de Setembro de 2005. Regulamento Técnico Para Produtos de Vegetais, Produtos de Frutas e Cogumelos Comestíveis.

[53] Szadzińska J., Mierzwa D. (2021). The influence of hybrid drying (microwave-convective) on drying kinetics and quality of white mushrooms. Chem. Eng. Process..

[54] Köprüalan Ö., Altay Ö., Bodruk A., Kaymak-Ertekin F. (2021). Effect of hybrid drying method on physical, textural and antioxidant properties of pumpkin chips. J. Food Meas. Charact..

[55] Islam M.Z., Das S., Monalisa K., Sayem A.S.M. (2019). Influence of osmotic dehydration on mass transfer kinetics and quality retention of ripe papaya (*Carica papaya* L.) during Drying. AgriEngineering.

[56] Shao L., Zhao Y., Zou B., Li X., Dai R. (2021). Ohmic heating in fruit and vegetable processing: Quality characteristics, enzyme inactivation, challenges and prospective. Trends Food Sci. Technol..

[57] Cao X., Zhang Y., Zhang F., Wang I., Yi J., Liao X. (2011). Effects of high hydrostatic pressure on enzymes, phenolic compounds, anthocyanins, polymeric color and color of strawberry pulps. J. Sci. Food Agric..

[58] Jafari F., Movagharnejad K., Sadeghi E. (2020). Infrared drying effects on the quality of eggplant slices and process optimization using response surface methodology. Food Chem..

[59] Mohammed H.H. (2022). Effect of Pretreatments and Solar Tunnel Dryer Zones on Drying Characteristics and Quality of Pumpkin (Cucurbita maxima) Pulp Slice and Powder. Master’s Thesis.

[60] Liu Y., Miao S., Wu J., Liu J., Yu H., Duan X. (2015). Drying characteristics and modeling of vacuum far-infrared radiation drying of *Flos lonicerae*. J. Food Process. Preserv..

[61] Djendoubi N.M., Boudhrioua N., Kechaou N., Courtis F., Bonazzi C. (2012). Influence of air drying temperature on kinetics, physicochemical properties, total phenolic content and ascorbic acid of pears. Food Bioprod. Process..

[62] Fonteles T.V., Costa M.G.M., Jesus A.L.T., Miranda M.R.A., Fernandes F.A.N., Rodrigues S. (2012). Power ultrasound processing of cantaloupe melon juice: Effects on quality parameters. Food Res. Int..

[63] Dyab A., El-El-Sherif G., Gab-Allah R. (2023). Evaluate the effect of pretreatments and drying techniques on the sweet potato slices. Food Technol. Res. J..

[64] Ramos-Parra P.A., García-Salinas C., Rodríguez-López C.E., García N., García-Rivas G., Hernández-Brenes C., Díaz de la Garza R.I. (2019). High hydrostatic pressure treatments trigger de novo carotenoid biosynthesis in papaya fruit (*Carica papaya* cv. Maradol). Food Chem..

[65] Kreck M., Kuerbel P., Ludwig M., Paschold P., Dietrich H. (2006). Identification and quantification of carotenoids in pumpkin cultivars (*Cucurbita maxima* L.) and their juices by liquid chromatography with ultraviolet-diode array detection. J. Appl. Bot. Food Qual..

[66] Rodriguez-Amaya D.B. (1999). Latin American food sources of carotenoids. Arch. Latinoam. Nutr..

[67] Rao A.V., Rao L.G. (2007). Carotenoids and human health. Pharmacol. Res..

[68] Mortensen A., Skibsted L.H. (2000). Kinetics and mechanism of the primary steps of degradation of carotenoids by acid in homogeneous solution. J. Agric. Food Chem..

[69] Veda S., Platel K., Srinivasan K. (2008). Influence of food acidulants and antioxidant spices on the bioaccessibility of β-carotene from selected vegetables. J. Agric. Food Chem..

[70] Ouyang M., Cao S., Huang Y. (2021). Phenolics and ascorbic acid in pumpkin (*Cucurbita maxima*) slices: Effects of hot air drying and degradation kinetics. Food Meas..

[71] Gonçalves E.M., Pinheiro J., Abreu M., Brandão T.R.S., Silva C.L.M. (2011). Kinetics of quality changes of pumpkin (*Curcurbita maxima* L.) stored under isothermal and non-isothermal frozen conditions. J. Food Eng..

[72] Arruda G.M.P., Brandão S.C.R., Silva Junior E.V., Silva E.M., Barros Z.M.P., Silva E.S., Shinohara N.K.S., Azoubel P.M. (2023). Influence of ultrasound and ethanol as a pretreatment on papaya infrared and convective drying characteristics and quality parameters. J. Food Process Eng..

[73] Carvalho G.R., Rojas M.L., Silveira I., Augusto P.E.D. (2020). Drying accelerators to enhance processing and properties: Ethanol, isopropanol, acetone and acetic acid as pre-treatments to convective drying of pumpkin. Food Bioprocess Technol..

[74] Onwude D., Hashim N., Janius R., Nawi N., Abdan E. (2017). Color change kinetics and total carotenoid content of pumpkin as affected by drying temperature. Ital. J. Food Sci..

[75] Lee F.A. (1983). Browning reactions. Basic Food Chemistry.

[76] Rizzi G.P. (1997). Chemical structure of colored Maillard reaction products. Food Rev. Int..

[77] Marquez-Rios E., Ocaño-Higuera V.M., Maeda-Martínez A.N., Lugo-Sánchez M.E., Carvallo-Ruiz M.G., Pacheco-Aguilar R. (2009). Citric acid as pretreatment in drying of Pacific Lion’s Paw Scallop (*Nodipecten subnodosus*) meats. Food Chem..

[78] Doymaz I. (2020). Impact of citric acid on the drying characteristics of kiwifruit slices. Acta Sci. Technol..

[79] Sun X., Jin X., Fu N., Chen X. (2020). Effects of different pretreatment methods on the drying characteristics and quality of potatoes. Food Sci. Nutr..

[80] Sahoo M., Titikshya S., Aradwad P., Kumar V., Naik S.N. (2022). Study of the drying behaviour and color kinetics of convective drying of yam (*Dioscorea hispida*) slices. Ind. Crops Prod..

[81] Seerangurayar T., Al-Ismaili A.M., Jeewantha L.J., Al-Habsi N.A. (2019). Effect of solar drying methods on color kinetics and texture of dates. Food Bioprod. Process..

[82] Xiao H.W., Yao X.D., Lin H., Yang W.X., Meng J.S., Gao Z.J. (2012). Effect of SSB (superheated steam blanching) time and drying temperature on hot air impingement drying kinetics and quality attributes of yam slices. J. Food Process Eng..

[83] Kumar Y., Sharanagat V.S., Singh L., Nema P.K. (2020). Convective drying of spine gourd (*Momordica dioica*): Effect of ultrasound pre-treatment on drying characteristics, color, and texture attributes. J. Food Process. Preserv..

[84] Sakooei-Vayghan R., Peighambardoust S.H., Hesari J., Peressini D. (2020). Effects of osmotic dehydration (with and without sonication) and pectin-based coating pretreatments on functional properties and color of hot-air dried apricot cubes. Food Chem..

